# Low bone turnover in premenopausal women with type 2 diabetes mellitus as an early process of diabetes-associated bone alterations: a cross-sectional study

**DOI:** 10.1186/s12902-017-0224-0

**Published:** 2017-11-29

**Authors:** Dyah Purnamasari, Melisa D. Puspitasari, Bambang Setiyohadi, Pringgodigdo Nugroho, Harry Isbagio

**Affiliations:** 1 Division of Metabolism and Endocrinology, Department of Internal Medicine, Faculty of Medicine Universitas Indonesia, Cipto Mangunkusumo Hospital, Jl. Salemba 6, Jakarta, 10430 Indonesia; 2 Department of Internal Medicine, Faculty of Medicine Universitas Indonesia, Cipto Mangunkusumo Hospital, Jakarta, Indonesia; 3 Division of Rheumatology, Department of Internal Medicine, Faculty of Medicine Universitas Indonesia, Cipto Mangunkusumo Hospital, Jakarta, Indonesia; 4 Division of Nephrology and Hypertension, Department of Internal Medicine, Faculty of Medicine Universitas Indonesia, Cipto Mangunkusumo Hospital, Jakarta, Indonesia

**Keywords:** T2DM, P1NP, CTX, Bone turnover, Premenopause, Diabetoporosis

## Abstract

**Background:**

Individuals with Diabetes Mellitus (DM) are at increased risk for fracture due to the decrease in bone strength and quality. Serum procollagen type I intact N-terminal (P1NP) and serum C-terminal cross-linking telopeptide of type I collagen (CTX) as markers of bone formation and resorption, respectively, have been reported to be decreased in T2DM. It remains unclear whether diabetes-associated alterations in the bone turnover of T2DM individuals are related to the longer duration of the disease or may occur earlier. Furthermore, previous studies on BTMs in T2DM individuals have mostly been done in postmenopausal women with T2DM, which might have masked the DM-induced alterations of bone turnover with concurrent estrogen deficiency. This study aims to assess the levels of serum P1NP and CTX as markers of bone turnover in premenopausal women with and without T2DM.

**Methods:**

This cross-sectional study involves 41 premenopausal women with T2DM, and 40 premenopausal women without DM. Sampling was done consecutively. P1NP and CTX measurement was done using the electrochemi-luminescence immunoassay (ECLIA) method. Other data collected include levels of HbA1C, ALT, creatinine, eGFR and lipid profile.

**Results:**

Median (interquartile range) P1NP in T2DM is 29.9 ng/ml (24.7–41.8 ng/ml), while in non-DM is 37.3 ng/ml, (30.8–47.3 ng/ml; *p* = 0.007). Median (interquartile range) CTX in T2DM is 0.161 ng/ml (0.106–0.227 ng/ml), while in non-DM is 0.202 ng/ml (0.166–0.271 ng/ml; *p* = 0.0035). Levels of P1NP and CTX in the T2DM group did not correlate with the duration of disease, age, BMI or the levels of HbA1C.

**Conclusions:**

Premenopausal women with T2DM indeed have lower bone turnover when compared with non-DM controls. This significantly lower bone turnover process starts relatively early in the premenopausal age, independent of the duration of DM. Gaining understanding of the early pathophysiology of altered bone turnover may be key in developing preventive strategies for diabetoporosis.

## Background

Diabetes mellitus (DM) is a chronic metabolic disorder with an increasing prevalence worldwide. Common late complications of DM are microvascular diseases including nephropathy, retinopathy, neuropathy, and macrovascular disease such as acute coronary syndrome. However, osteoporotic fracture is increasingly recognized as an important complication in both men and women with type 1 DM (T1DM) and type 2 DM (T2DM). Diabetic osteopathy, or may also termed as “diabetoporosis” [1], is diabetes-associated bone alterations that are characterized by a decrease in bone quality, leading to an increased risk of bone fracture in both types of DM [2, 3]. A meta-analysis of 5 studies reported that T1DM is associated with an overall relative risk (RR) of 8,9 (95% CI 7,1–11,2) for hip fractures when compared with an age-matched nondiabetic population [4]. Similarly, adults with T2DM have a 50%–80% higher risk of hip fractures [5, 6] as well as of extremity fractures [5, 7].

While individuals with T1DM showed decreased bone mass density (BMD) [8–13], T2DM is often characterized by increased or unchanged BMD [5, 14–18]. Thus, bone fragility in T2DM depends on microarchitectural changes that is largely determined by increased cortical porosity in T2DM individuals compared to non-diabetic controls rather than the reduction in bone mineral mass [19–22]. The mechanisms of DM-induced bone fragility in T1DM and T2DM are complex and only partially overlap [23]. It was thought that β-cell failure and low levels of IGF1 disrupt osteoblasts function during growth in T1DM, resulting in low peak bone mass at a young age [24]. In contrast, glucose toxicity, advanced glycation end-products (AGEs), cytokines and adipokines that are affecting osteocyte, bone turnover and collagen affect T2DM individuals at a later age [25].

Data from the study of De Liefde et al. suggested an association between the duration of T2DM and the risk of fractures, as the increased fracture risk found in subjects with DM was restricted to subjects with already established and treated DM only [26]. However, it remains unclear whether changes in bone turnover of T2DM individuals are related to the longer duration of disease or may occur earlier. Data on altered bone metabolism in T2DM have mostly included older, postmenopausal women, that did not make the distinction between T1DM and T2DM or between men and women. Furthermore, estrogen is an important determinant of bone health, which dramatically decreases upon menopause [27]. It is not well-established whether bone fragility in diabetes mirror those found in primary osteoporosis, which occurs frequently in postmenopausal women, but in different proportions, or whether there were particular alterations in the bone of individuals with DM that are not found in primary osteoporosis [1]. Regardless, clinical studies performed on postmenopausal women with T2DM would not be able to exclude the confounding effect of estrogen deficiency on bone metabolism in T2DM. Only one prior study examined BMD and bone turnover markers in both pre- and post-menopausal women with DM. Although showing that post-menopausal women had lower BMD compared with pre-menopausal women, this study found no difference in the levels of bone formation marker in the pre-menopausal women when compared to the reference values, as measured using osteocalcin [28].

Changes in bone turnover can be asessed by measurements of serum levels of C-terminal cross-linking telopeptide of type I collagen (CTX) and procollagen type I intact N-terminal (P1NP), which have been recommended as markers of bone resorption and formation, respectively [29]. The early pathophysiology of altered bone turnover in T2DM remains largely unknown, and informative data may aid in developing preventive strategies for diabetoporosis. To the best of our knowledge, no study has provided data on whether BTM in premenopausal T2DM women is altered. This study aimed to assess whether changes in bone turnover occur early in T2DM by measuring levels of the bone resorption and formation markers, CTX and P1NP, in premenopausal women with T2DM.

## Methods

### Study population

We performed a cross-sectional study enrolling 81 premenopausal women aged >35 years: 41 subjects with T2DM and 40 subjects without diabetes to assess the levels of serum P1NP and CTX as markers of bone turnover in premenopausal women. From April to August 2017, we consecutively recruited subjects from the outpatient clinic in several hospitals in Jakarta (Cipto Mangunkusumo Hospital, Persahabatan Hospital, Tugu Koja Hospital and Kemayoran Hospital). All T2DM individuals had been diagnosed according to American Diabetes Association criteria (2016) for at least 5 years. All subjects were malay in race, and had neither hepatic, gastrointestinal, or thyroid diseases nor other secondary causes for low BMD. None of the subjects had been treated with steroid for more than 3 months in the past 3 years, hormone therapy or hormonal contraception, bisphosphonates, antipsychotic drugs, anticonvulsant drugs, hydrochlorothiazides, or thiazolidinediones that might affect bone mass. Subjects with stage 4 and 5 chronic kidney disease and BMI lower than 18.5 were excluded.

### Biochemical measurements

Samples of venous blood were collected in EDTA-anticoagulated tubes (BD Vacutainer, Becton Dickinson) in the morning after fasting overnight. Samples were centrifuged immediately after collection at 4000 g for 8 min. Plasma were aliquoted and stored at −80 °C until examination. Serum levels of CTX (detection range: 0,010–6 ng/ml) and P1NP (detection range: 5–1200 ng/ml) were measured by using automated electrochemiluminescent sandwich antibody assay (ECLIA) on Cobas® analyzer (Roche Diagnostics, Manheim, Germany).

### Ethical aspects

The study was approved by the Health Research Ethics Committee of University of Indonesia and Cipto Mangunkusumo Hospital, Jakarta, Indonesia. All participants provided written informed consent to participate.

### Statistical analyses

Data were expressed as median and interquartile range (IQR). Values in groups were compared by T-test or Mann Whitney U test depending on the distribution of the variable of interest. The Pearson (for normally distributed data) or Spearman correlation coefficient (for non-normally distributed data) was used for analyses of correlations. All analyses were performed with Graphpad Prism version 7 (San Diego, CA, USA). *P* < 0.05 were considered statistically significant.

## Results

### Subject characteristics

Clinical characteristics for the study groups are shown in Table 1. All subjects included in this study were premenopausal women. T2DM individuals had higher median age (interquartile range, IQR; 45, 41–49 years vs. 39, 37–45 years, *p* = 0.0004) than non-diabetic controls. Median HbA1c levels of the T2DM group were 10% (7.7–11.6), and median T2DM duration was 9 years (5–12 years). Fourteen (34%) of the T2DM individuals were on insulin treatment, 7 on metformin alone and 11 on a combination of metformin and sulfonylurea while one did not use any treatment. There were 12 T2DM subjects and 7 non-diabetic controls with BMI > =30. None of the subjects consume alcohol or smoke. That CTX is excreted in the urine is an important consideration for studies in T2DM which involves patients with chronic kidney disease, which have been excluded from our study.

**Table 1:** Characteristics of subjects

	T2DM	Non-DM Controls
Number	41	40
Age (years)	45 (41–49)	39 (37–45)
Duration of DM (years)	9 (5–12)	NA
BMI (kg/m^2^)	26 (23–31)	25 (23–29)
HbA1C (%)	10 (7.7–11.6)	5.5 (5.2–5.8)
Creatinine (mg/dl)	0.6 (0.5–0.8)	0.7 (0.6–0.7)
eGFR (mL/min/1,73 m^2^)	106 (87–115)	110 (106–116)
SGPT (U/l)	19 (11–25)	16 (11–21)
Triglycerides (mg/dl)	158 (96–198)	ND
LDL (mg/dl)	141 (117–163)	ND
HDL (mg/dl)	49 (38–54)	ND
Total cholesterol (mg/dl)	197 (179–230)	ND
Antidiabetics		
- Metformin (%)	7 (17)	NA
- Metformin + Sulfonylurea (%)	11 (27)	NA
- Insulin (%)	14 (34)	NA
- Combined therapy (%)	8 (20)	NA
- No treatment (%)	1 (2)	NA

Data depicted are median with interquartile range (IQR) unless otherwise indicated. Statistical differences are analyzed by using T-test or Mann-Whitney U test. NA, not applicable; ND, not determined

### Premenopausal women with T2DM had lower bone turnover than non-DM controls

**Fig. 1:**
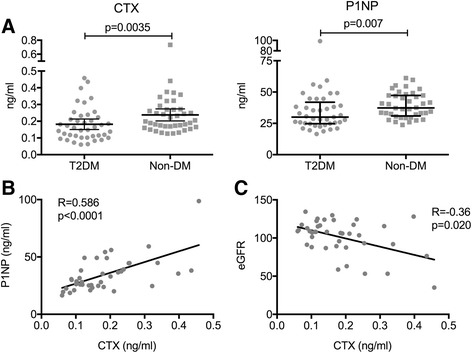
Lower bone turnover in premenopausal women with T2DM compared to controls. **a** Individuals with T2DM had lower levels of CTX (left panel) and P1NP (right panel) when compared to non-DM controls. **b** Positive correlation was found between P1NP and CTX level, **c** Negative correlation was found between CTX level and eGFR

T2DM individuals demonstrated a significantly lower CTX, a marker of bone resorption, and P1NP, a marker of bone formation. Median values of CTX in the T2DM group were 0.161 ng/ml (0.106–0.227 ng/ml), lower when compared to the non-diabetic group (0.202 ng/ml, 0.166–0.271 ng/ml; *p* = 0.0035; Fig. 1). Similarly, P1NP levels were also lower in the T2DM subjects (29.9 ng/ml, 24.7–41.8 ng/ml) compared to the non-diabetic (37.3 ng/ml, 30.8–47.3 ng/ml; *p* = 0.007). Levels of CTX and P1NP correlated positively with one another (Spearman *R* = 0.586, *P* < 0.0001). CTX is excreted in the urine, and CTX levels in the T2DM subjects negatively correlated with estimated glomerular filtration rate (eGFR; *R* = −0.36, *p* = 0.02), whereas this correlation was not observed for P1NP (*R* = −0.17, *p* = 0.28).

### Correlations between bone turnover markers with age and different clinical parameters

**Fig. 2:**
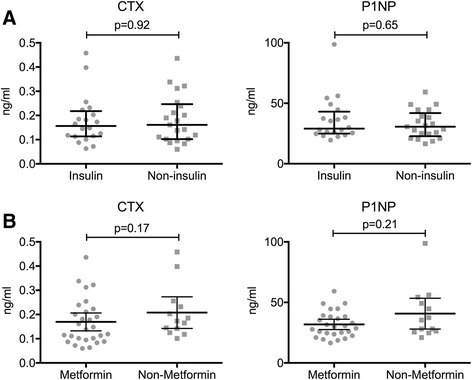
There was no correlation between insulin or metformin use with bone turnover in T2DM. **a** Spearman correlation of CTX levels and eGFR in T2DM individuals. **b** CTX (upper and lower left panels) and P1NP levels (upper and lower right panels) in individuals with and without insulin or metformin use

There were no statistically significant correlations between CTX and P1NP levels of T2DM subjects with age (*p =* 0.38 and *p =* 0.66*)*, duration of DM (*p* = 0.76 and *p* = 0.12) and levels of HbA1C (*p* = 0.26 and *p* = 0.27; Fig. 2). We also did not find correlations between the BTMs and LDL, HDL, total cholesterol, or BMI. Median CTX and P1NP levels were comparable in subjects with or without insulin (CTX: 0.16 ng/ml, 0.11–0.21 ng/ml vs. 0.16 ng/ml, 0.10–0.24 ng/ml, *p* = 0.92; P1NP: 29 ng/ml, 25–43 ng/ml vs. 30.6 ng/ml, 22.8–41.9 ng/ml, *p* = 0.65), as well as in those with or without metformin (CTX: 0.14 ng/ml, 0.9–0.22 ng/ml vs. 0.17 ng/ml, 0.13–0.24 ng/ml, *p* = 0.17; P1NP: 30 ng/ml, 23.8–38.2 ng/ml vs. 35.4 ng/ml, 25.9–51.8 ng/ml, *p* = 0.20).

## Discussion

Our study reveals three important findings. First, women with T2DM indeed have lower bone turnover when compared with non-DM controls. Second, this significantly lower bone turnover process in T2DM women starts early in the premenopausal age independent of the duration of DM. Third, we did not find any difference in the levels of BTMs between those with or without insulin or metformin use. To the best of our knowledge, this is the first study measuring bone turnover markers in premenopausal women with T2DM. Our findings are in agreement with previous studies which reported lower bone resorption [30–38] and bone formation [35, 39] in T2DM individuals.

T2DM, in contrast with T1DM, is interestingly characterized by increased or unchanged bone mineral density (BMD) [5, 14–18]. It is therefore the severe deficit in bone quality and changes to the bone micro-architecture, as depicted by increased bone cortical porosity T2DM, that underlies fragility fractures in T2DM [19, 30, 40, 41]. Despite their lower bone turnover, T2DM individuals have increased bone fragility that cannot be captured by measuring BMD alone [39, 42]. Data showed that the higher BMD in T2DM was not associated with bone geometrical instability or bending strength [43]. However, a study by Burghardt and colleagues used a high-resolution peripheral quantitative computed tomography that enabled assessment of volumetric BMD (vBMD) independently in cortical and trabecular compartments of the bone. T2DM subjects, especially those with previous fracture, showed marked levels of intracortical porosity with an extremely dense trabecular bone in the peripheral region adjacent to the cortex, an assessment that is not captured by conventional measurement of BMD. The authors concluded that this a potential explanation for the inability of BMD measurements to explain increased fracture incidence in patients with T2DM [22]. Another study also reported lower cortical vBMD in T2DM individuals [44]. An alternative explanation for the increased BMD seen in T2DM individuals is the relatively heightened state of bone mineralization as a result of a slowing down in the process of replacing older, more densely mineralized bone with younger, less densely mineralized bone [42].

The duration of T2DM is thought to contribute to the development of bone fragility in T2DM [26]. This notion is supported by studies reporting the association between fracture risk and diabetic microvascular complications such as retinopathy [15], neuropathy [45] and cerebrovascular diseases [46]. It is also thought that long-term DM individuals suffer from more DM-related complications which consequently increase their risk for falling [26]. Furthermore, hyperglycemia which occurs for an extended period of time induces higher levels of AGEs in the bone collagen [47, 48], which negatively correlated with the material and biomechanical properties of both the cortical and cancellous bone [49]. AGEs, which are markedly increased in T2DM individuals [50], are diverse compounds generated through the non-enzymatic glycation or glycoxidation of proteins, lipids, and nucleic acids. AGEs interfere with normal osteoblast function, attachment to the collagen matrix, and impair osteoblast development. AGEs may also decrease bone resorption by alteration of the structural integrity of matrix proteins and inhibiting the osteoclastic differentiation. AGEs crosslinking in collagen also leads to more brittle bones that are less able to deform before fracturing [51]. CTX is the degradation product of type I collagen [29] and is used in numerous studies in T2DM as a marker of bone resorption [52]. and there is the possibility that the degraded type I collagen might have already been glycated in diabetic patients. Despite this, to date, there is no method currently available that can differentially measure glycated CTX from unglycated CTX.

That insulin and metformin usage in our study was not associated with levels of BTMs were not surprising. Although fractures were shown to be increased in T2DM individuals treated with insulin [53], it appeared that the more aggressive glycemic control in elderly individuals with long term disease might increase microvascular complications, such as diabetic retinopathy, and hypoglycemic events and thus the risk for falls and fractures [54]. Metformin, the first line drug for DM, was previously found from most clinical studies to have positive or neutral effect on BMD and fracture risk in large cohorts [55–57].

Whether individuals with T2DM has bone alterations that are specific for T2DM, or whether it is similar to primary osteoporosis but occur in different proportions have not yet been well-established [1]. Interestingly, our data also revealed that changes in the bone turnover of these subjects reveal, at least clinically, a profile that is more similar to those associated with T2DM, with decreased bone resorption, rather than with increasing age or primary osteoporosis, where increased bone resorption is often seen [58]. We did not find any association between levels of CTX and P1NP with the duration of DM or HbA1C levels, as previously reported in other studies [33, 59, 60]. A study by Kanazawa et al. indicated that it was changes in HbA1C during glycemic control, rather than baseline HbA1C, that was associated with changes in bone formation [61], which might explain our lack of correlation between the BTMs and HbA1C in our study. This study is performed in the Indonesian population with all subjects being of Malay ethnicity. Some studies indicate that there were inherent differences in bone turnover between the different ethnicities [62–67]. However, others suggest that ethnicity per se is not important, and that apparent ethnic differences in rates of bone loss were largely explained by differences in body weight [29, 68, 69]. Studies also reported that levels of P1NP and CTX may be influenced by BMI [70], of which did not differ among groups in our study. Gender differences in BMD, bone structure and the risk of fracture in individuals with diabetes have been acknowledged, with women being particularly at risk for DM-associated bone alterations [71]. It remains largely unclear to what extent hypoglycaemic drugs are gender-specific. Only thiazolidinediones were reported to have negative effects on bone metabolism in women [72–74]. Although our study only involved female subjects, there were none who were on thiazolidinediones.

Better knowledge on how DM and its treatments influence bone tissue may be the basis of effective prevention of fragility fractures in individuals with DM. Currently, recommendations for diabetoporosis management include glycemic control, adequate intake of calcium and vitamin D, screening for low BMD, and prevention and treatment of diabetic complications [75]. Our study emphasizes that changes in bone metabolism occur early and may warrant preventive efforts or pharmacological interventions when needed, although this needs well-designed clinical trials of anti-osteoporotic drugs specifically for the diabetic population.

The limitations of our study include the cross-sectional design which measures the investigated parameters in a single time point, and the small sample size of this study. Furthermore, data on BMD from dual energy x-ray absorptiometry is not available, limiting our assessment into the relationship between early alterations of the bone turnover markers and bone mineralization. Lastly, data on hormonal status of these subjects were also not available, and would have provided information on the correlation between hormonal status and bone metabolism in the premenopausal women. Despite the above limitations, this study remains as the only study to date revealing a decrease in bone turnover markers in premenopausal women with T2DM.

## Conclusion

Premenopausal women with T2DM had lower levels of CTX and P1NP, which reflects lower bone turnover, compared to non-DM controls, independent of age and duration of disease. Our results indicate that alterations in bone turnover occur relatively early in the course of the disease, and prior to the occurrence of menopause. As T2DM individuals are at increased risk of fracture, understanding the early pathophysiology of altered bone turnover may be key in developing preventive strategies for diabetoporosis.

## Abbreviations

AGE: advanced glycation endproducts
BMD: bone mineral density
BMI: body mass index
BTM: bone turnover marker
CTX: C-terminal cross-linking telopeptide of type I collagen
DM: diabetes mellitus
eGFR: estimated glomerular filtration rate
HDL: high density lipoprotein
LDL: low density lipoprotein
P1NP: procollagen type I intact N-terminal (P1NP) and serum
T1DM: type 1 diabetes mellitus
T2DM: type 2 diabetes mellitus
